# Precision-Cut Liver Slices: A Valuable Preclinical Tool for Translational Research in Liver Fibrosis

**DOI:** 10.3390/ijms26167780

**Published:** 2025-08-12

**Authors:** Meritxell Perramón, Manuel Macías-Herranz, Rocío García-Pérez, Wladimiro Jiménez, Guillermo Fernández-Varo

**Affiliations:** 1 Biochemistry and Molecular Genetics Service, Hospital Clínic of Barcelona, Institut d’Investigacions Biomèdiques August Pi i Sunyer (IDIBAPS), 08036 Barcelona, Spain; meri.perramon@gmail.com (M.P.); mmaciash@recerca.clinic.cat (M.M.-H.); wjimenez@clinic.cat (W.J.); 2 Centro de Investigación Biomédica en Red de Enfermedades Hepáticas y Digestivas (CIBERehd), ISCIII, 28029 Madrid, Spain; 3 Department of Biomedicine, University of Barcelona, 08036 Barcelona, Spain; 4 Hepatopancreatobiliary Surgery & Transplantation, General & Digestive Surgery Service, Digestive & Metabolic Disease Institute (ICMDM), Hospital Clínic of Barcelona, 08036 Barcelona, Spain; rgarcia5@clinic.cat; 5 Department of Medicine, University of Barcelona, 08036 Barcelona, Spain

**Keywords:** translational liver research, ex vivo model of liver fibrosis, preclinical tools, rat and human precision cut liver slices, hyperoxia, pathological gene signature

## Abstract

Halting liver fibrosis progression is a key goal in treating liver disease, yet effective antifibrotic drugs remain unavailable. This study explores the use of precision-cut liver slices (PCLS) as an ex vivo model to evaluate new therapies. Researchers tested how different oxygen levels affect viability, tissue integrity, and inflammatory response in PCLS from healthy and fibrotic rats. Fibrotic PCLS maintained their pathological gene signature under 40% oxygen and responded to inflammatory stimuli, indicating preserved functionality. Exposure to high oxygen levels increased oxidative stress and pro-inflammatory gene expression. Cirrhotic PCLS showed early signs of reduced viability and the upregulation of fibrosis-related genes including *Col1α2*, *Col3α1*, *αSMA*, *Timp1*, *Timp2*, *Mmp2*, *Pdgfrβ*, *Nos2*, *Cox2*, and *Il6*. Lipopolysaccharide (LPS) exposure induced the marked overexpression of *Nos2* and *Il1β* mRNA and confirmed the model’s responsiveness to external injury. Fibrotic PCLS retained fibrogenic activity for at least 48 h. Additionally, the adenoviral delivery of a dominant-negative soluble PDGFRβ effectively blocked fibrotic signaling. Human fibrotic PCLS also remained viable for 72 h and showed an increased mRNA expression of fibrosis markers such as *COL1A1*, *αSMA*, and *MMP2*. These results highlight the potential of PCLS as a promising platform for future therapeutic testing, pending further validation with functional interventions.

## 1. Introduction

Liver diseases have become a first-order health problem in Western countries. The burden of these diseases in Europe is the largest in the world and continues to grow [1]. Different factors leading to hepatic dysfunction explain this phenomenon. Among these factors, the most relevant are harmful alcohol consumption, obesity, and viral hepatitis.

Fibrosis results from the chronic injury of the hepatic parenchyma and consists of very active extracellular matrix (ECM) remodeling with the progressive and abundant deposition of collagen fibers. Since cirrhosis is a major determinant of morbidity and mortality, halting the progression of fibrosis to cirrhosis has largely been considered as a foremost goal in patients with liver disease. Several promising therapeutic candidates have been identified to halt or reverse hepatic fibrogenesis, including agents that target specific molecular pathways involved in HSC activation, anti-inflammatory agents, renin angiotensin system inhibitors, cannabinoid receptor antagonists, or PDGF antagonists [2,3,4,5]. However, most of these compounds have shown limited efficacy and/or adverse side effects. In fact, there is currently only one FDA-approved therapy to halt the progression of liver fibrosis in patients with metabolic dysfunction-associated steatotic liver disease (MASLD) [6]. Despite this, an approved pharmacological treatment that directly and consistently targets liver fibrosis remains lacking. One of the major challenges in overcoming this issue is the need to bridge the gap between the results obtained in cell cultures or in vivo animal models of fibrosis and those achieved when new drugs are tested in a clinical setting.

Precision-cut liver slices (PCLS) are a unique ex vivo model that closely mimics the in vivo environment. They maintain the complex architecture and cellular diversity of the liver, allowing for the study of liver functions and disease mechanisms in a context that closely resembles the in vivo situation [7,8,9]. Moreover, PCLS can be prepared from human liver tissue, offering a relevant model for studying human-specific liver diseases and drug responses [10,11,12]. This enhances the predictive value of preclinical findings and facilitates the development of effective therapies for human patients.

**Figure 1:**
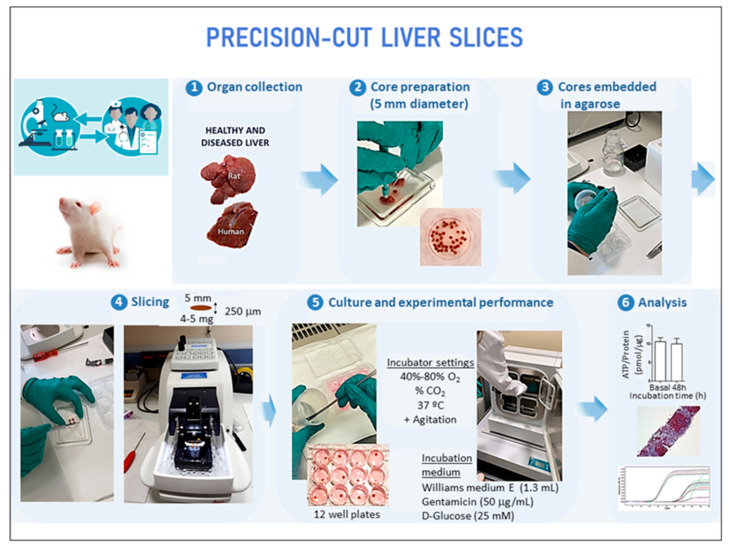
Schematic diagram of the experimental design. Sequence of the procedures performed in the study: organ collection (1), core preparation (2), cores embedded in agarose (3), slicing (4), culture and experimental performance (5), and analysis (6).

This investigation aimed to assess whether PCLS accurately reflect the molecular changes occurring during the fibrogenic process and whether this system could become a useful preclinical tool to predict the behavior of antifibrotic drugs in patients with liver disease (Figure 1).

## 2. Results

Table 1 shows the serological parameters of all of the control and carbon tetrachloride (CCl_4_)-treated rats included in the study. As expected, fibrotic rats exhibited a significant activation of hepatic enzymes, elevated serum bilirubin levels, and notably reduced serum concentrations of total proteins, albumin, glucose, and triglycerides. These disturbances are characteristic of advanced liver disease.

### 2.1. Hyperoxia, Cell Viability, Tissue Integrity, and mRNA Expression of Inflammatory Mediators in Cirrhotic and Control rPCLS

**Figure 2:**
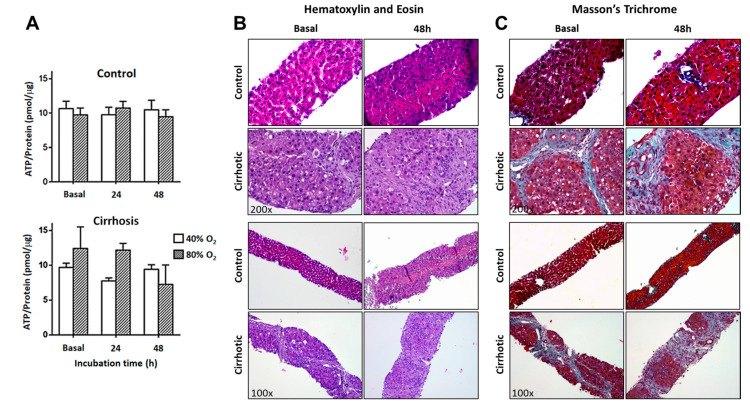
Effect of varying O_2_ concentrations on viability and tissue integrity in rPCLS from control and cirrhotic rats. Viability was assessed using the ATP/protein ratio (**left panel**) on freshly cut slices (basal conditions) and under oxygen concentrations ranging from 40% to 80% for up to 48 h (**A**). Liver sections from control and cirrhotic rats were stained with hematoxylin and eosin (H&E) and Masson’s trichrome to evaluate tissue architecture and fibrosis progression following chronic CCl_4_ inhalation ((**B**,**C**); magnification: 100× and 200×). Experiments were conducted on freshly cut slices (basal conditions) and at an O_2_ concentration of 40% for up to 48 h.

Tissue oxygenation represents a critical condition for PCLS viability [9]. Due to the lack of vascular perfusion in these ex vivo tissue sections, experiments were conducted under hyperoxic conditions to ensure adequate oxygen diffusion. Here we assessed whether the intensity of the O_2_ exposure affects viability and tissue integrity. The experiments were conducted at between 40 and 80% O_2_ for up to 48 h, as performed in most previous studies. Freshly cut slices were included and considered as basal conditions. No alterations in viability or tissue integrity were observed when the experiments were performed within this range of conditions (Figure 2). The qualitative assessment of H&E-stained sections at higher magnification revealed that the control PCLS showed preserved lobular architecture in most regions, with limited signs of structural disruption. Cirrhotic livers exhibited severe architectural disruption, with bridging fibrous septa linking portal tracts and central veins. Hepatocytes displayed centrilobular necrosis, identified by structural loss and nuclear fragmentation (Figure 2B). Masson’s trichrome staining revealed minimal collagen content in the controls, while cirrhotic PCLS showed dense septa enclosing regenerative nodules, indicating chronic injury and fibrosis (Figure 2C).

**Figure 3:**
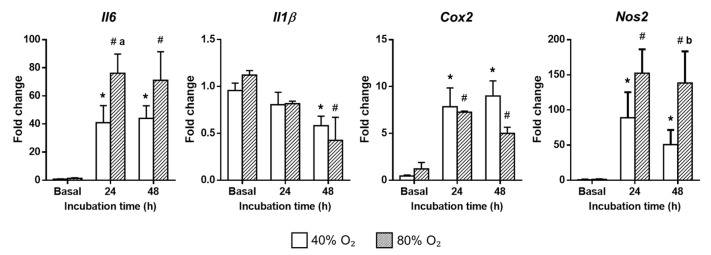
Impact of hyperoxia on hepatic mRNA expression of *Il6*, *Il1β*, *Cox2*, and *Nos2* in cirrhotic rPCLS. Samples were incubated under 40% to 80% O_2_ for up to 48 h under basal conditions. Gene expression was analyzed after 24 and 48 h of incubation. * *p* < 0.05 vs. Basal 40% O_2_; ^#^ *p* < 0.05 vs. Basal 80% O_2_; ^a^ *p* < 0.05 vs. 24 h 40% O_2_; ^b^ *p* < 0.05 vs. 48 h 40% O_2_. One-way ANOVA and Tukey’s multiple comparison test or Kruskal–Wallis’s and Dunn’s multiple comparison test were used when appropriate. Results were shown as mean ± S.E.

In contrast, hyperoxia markedly induces the expression of *Il6*, *Cox2*, and *Nos2* mRNA in rPCLS. This phenomenon is observed in both the control and cirrhotic PCLS. However, the oxidative stress induced by hyperoxia is significantly more intense when O_2_ concentrations reach their highest levels. In fact, an O_2_ concentration of 80% results in a significantly increased abundance of *Il6* and *Nos2* transcripts after 24 h and 48 h, respectively, which are widely recognized biomarkers of inflammatory response. These findings were associated with a temporal decrease in *Il1β* mRNA levels, a key pro-inflammatory cytokine that peaks during the early inflammatory phase and declines as fibrosis progresses (Figure 3).

### 2.2. Fibrotic rPCLS Maintain Their Pathological Gene Signature and Respond to Lipopolysaccharide (LPS) Exposure

Although cirrhotic liver tissue already exhibits an elevated expression of these genes at baseline, the slicing and short-term culture process can influence gene expression, particularly under high oxygen tension. Therefore, confirming that fibrotic gene expression remains elevated under 40% O_2_ after one hour of culture is essential to validate the stability and translational relevance of the model. To determine whether modulating oxygen levels to 40%—a condition that better preserves tissue viability while minimizing inflammation—still maintains the molecular expression patterns of genes involved in extracellular matrix remodeling, inflammation, or growth factors, we assessed the messenger RNA expression of a panel of transcripts. These transcripts included *Col1α2*, *Col3α1*, *αSMA*, *Timp1*, *Timp2*, *Mmp2*, *Mmp9*, *Pdgfrβ*, *Tgfβr1*, *Nos2*, *Cox2*, *Il1β*, *Tnfα*, and *Il6*. The measurements were performed using RT-PCR in both the control and fibrotic rPCLS, after a 1 h incubation at an O_2_ concentration of 40%. Compared with the rPCLS obtained from healthy animals, the fibrotic rPCLS displayed slightly impaired tissue viability alongside a wide induction of genes related to extracellular matrix (ECM) remodeling. This was particularly notable in the mRNA abundance of *Col1α2*, *Col3α1*, and *Mmp2* (Table 2). Additionally, there was a significant activation of pro-inflammatory genes such as *Nos2*, *Cox2*, and *Il6*. These findings support the concept that despite the considerable stress exerted during the preparation of the PCLS, they maintain most of the relevant molecular alterations present in the fibrotic liver.

**Figure 4:**
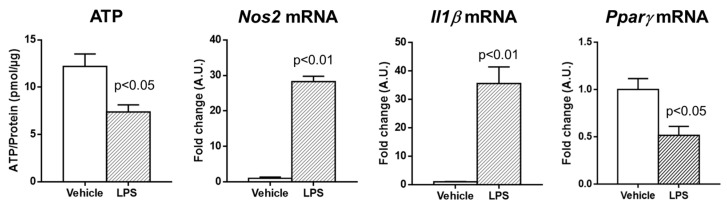
Effect of lipopolysaccharide (LPS) exposure on cirrhotic rPCLS. LPS treatment (10 µg/mL) was applied during the first hour of culture and was limited to a single one-hour exposure. Tissue viability was measured via the ATP/protein ratio. Hepatic mRNA expression of *Nos2*, *Il1β,* and *Pparγ* was assessed using RT-PCR following a 1 h incubation at 40% O_2_. Unpaired *t*-test was used. Results are given as means ± S.E.

Next, to assess whether rPCLS can respond to external sources of tissue injury, the slices were exposed to lipopolysaccharide (LPS), a well known inducer of inflammatory response in liver tissue. The results shown in Figure 4 indicate that LPS moderately compromised tissue viability but also resulted in an intense pro-inflammatory response, clearly reflected in the marked overexpression of *Nos2* and *Il1β* mRNA and the significantly decreased expression of *Pparγ* mRNA levels.

### 2.3. Time Course mRNA Expression of Genes Involved in ECM Remodeling

**Figure 5:**
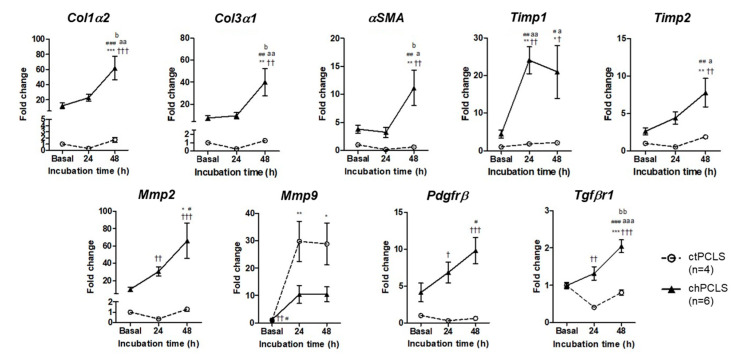
Cirrhotic PCLS present a clearly differentiated pattern of the expression of genes involved in profibrogenic metabolic pathways compared with control PCLS and these differences are maintained for up to 48 h. * *p* < 0.05, ** *p* < 0.01, *** *p* < 0.001 vs. Basal ctPCLS; ^†^ *p* < 0.05, ^††^ *p* < 0.01, ^†††^ *p* < 0.001 vs. 24 h ctPCLS; ^#^ *p* < 0.05, ^##^ *p* < 0.01, ^###^ *p* < 0.001 vs. 48 h ctPCLS; ^a^ *p* < 0.05, ^aa^ *p* < 0.01, ^aaa^ *p* < 0.001 vs. Basal chPCLS; ^b^ *p* < 0.05, ^bb^ *p* < 0.01 vs. 24 h chPCLS. One-way ANOVA and Tukey’s multiple comparison test or Kruskal–Wallis’s and Dunn’s multiple comparison tests were used when appropriate. Results were shown as mean ± S.E.

To ascertain whether the PCLS maintain their molecular signature over extended periods of time, rPCLS were incubated for up to 48 h. The mRNA expression patterns of an extensive and representative panel of genes were assessed, including *Col1α2* and *Col3α1*, *αSMA*, *Timp1*, *Timp2*, *Mmp2,* and *Mmp9*. The messengers of *Pdgfrβ* and T*gfβr1* were also evaluated (Figure 5). The rPCLS of the control and cirrhotic animals exhibited marked differences in the mRNA expression of all these genes. While almost no changes were observed in the control rPCLS during the 48 h incubation period, rPCLS from cirrhotic rats displayed a significant activation of all these transcripts under basal conditions. Furthermore, they showed a time-dependent increase in mRNA abundance, reaching peak values at 48 h. These findings indicate that under our experimental conditions, the fibrogenic activation of cirrhotic rPCLS is maintained for at least 48 h.

### 2.4. Infection with Adenovirus-Encoding Soluble PDGFRβ Abrogates the PDGFRβ Signaling Pathway in rPCLS

**Figure 6:**
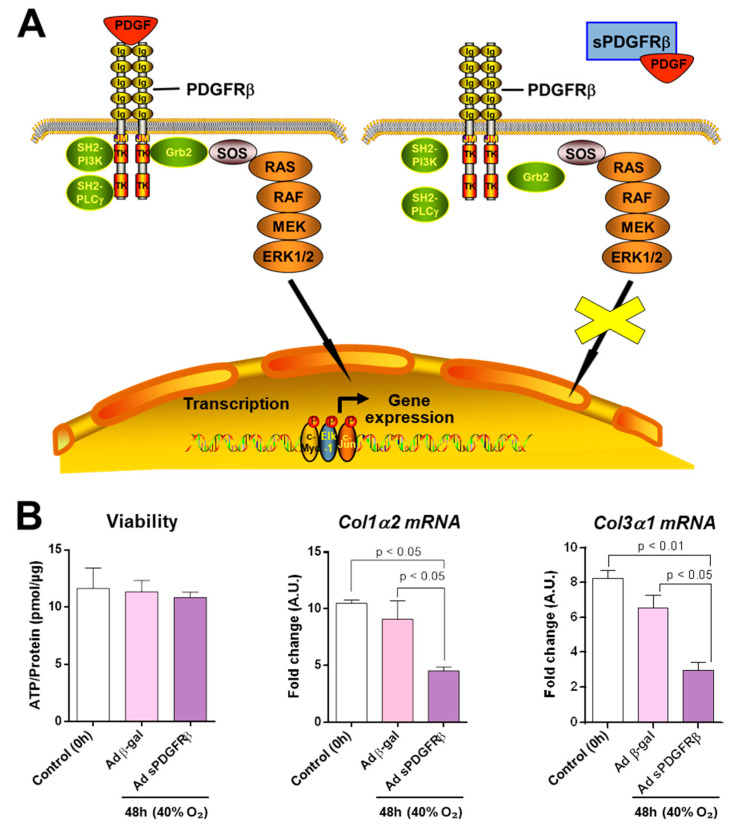
PCLS as an alternative model for evaluating therapeutic efficacy in liver tissue. Panel (**A**) illustrates the inhibition of the *PDGFRβ* signaling pathway by sPDGFRβ. Panel (**B**) shows the effects of adenoviral infection with Ad-sPDGFRβ or Ad-β-gal (control), both at 1000 m.o.i. The white bars in the graphs represent the basal condition (0 h) of rPCLS, which serves as the reference point for relative gene expression. After 48 h of incubation at 40% O_2_, tissue samples were collected to assess the viability and hepatic expression of *Col1α2* and *Col3α1.* One-way ANOVA and Tukey’s multiple comparison test or Kruskal–Wallis’s and Dunn’s multiple comparison tests were used when appropriate. Results were shown as mean ± S.E.

Next, we aimed to evaluate whether PCLS can serve as an ex vivo platform to investigate the effectiveness of antifibrogenic treatments in liver fibrosis. Therefore, we infected cirrhotic rPCLS with an adenoviral dominant-negative form of *PDGFRβ*. Previous studies by our laboratory and others have shown that the intravenous administration of this adenoviral construct in vivo interferes with the *PDGF* signaling pathway, leading to a reduction in fibrosis in CCl_4_-treated rats. We used two replication-defective recombinant adenoviral constructs expressing a dominant-negative soluble PDGFRβ (sPDGFRβ), which encodes the extracellular domain of PDGF receptor type β, or β-gal. rPCLS infected with either construct showed no alteration in viability, as indicated by adenosine triphosphate (ATP) content at the end of the study. No significant changes in the mRNA expression of *Col1α2* and *Col3α1* were observed in samples infected with the adenoviral construct containing β-gal. However, infection with adenovirus-encoding sPDGFRβ significantly reduced the mRNA expression of both *Col1α2* and *Col3α1* in fibrotic rPCLS (Figure 6). These results support the concept that PCLS could represent an alternative method to evaluate the efficacy of new treatments in liver tissue.

### 2.5. mRNA Expression of Fibrosis-Related Genes and Tissue Viability of Non-Fibrotic and Fibrotic hPCLS

**Figure 7:**
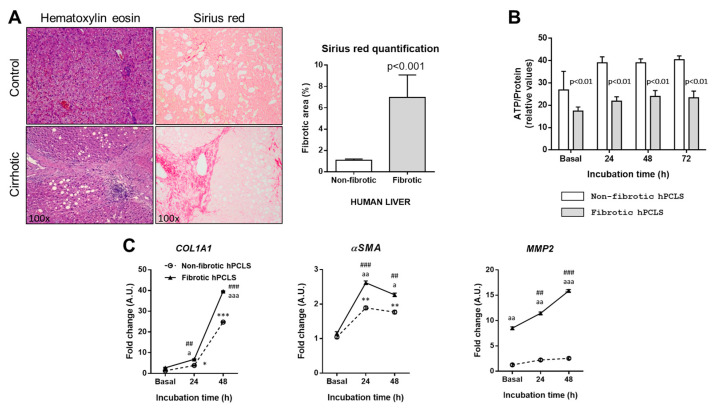
Representative images of H&E and Sirius Red staining in liver sections (100×) of non-fibrotic and fibrotic hPCLS, and corresponding morphometric quantification of Sirius Red (**A**). Tissue viability of non-fibrotic and fibrotic hPCLS incubated in basal conditions and at an O_2_ concentration of 40% up to 72 h (**B**). Hepatic mRNA expression of *COL1A1*, *αSMA*, and *MMP2* in non-fibrotic and fibrotic hPCLS incubated in basal conditions and at an O_2_ concentration of 40% up to 48 h (**C**). * *p* < 0.05, ** *p* < 0.01, *** *p* < 0.001 vs. basal value non-fibrotic hPCLS; ^##^ *p* < 0.01, ^###^ *p* < 0.001 vs. basal value fibrotic hPCLS; ^a^ *p* < 0.05, ^aa^ *p* < 0.01, ^aaa^ *p* < 0.001 vs. non-fibrotic hPCLS. One-way ANOVA and Tukey’s multiple comparison test or Kruskal–Wallis’s and Dunn’s multiple comparison tests were used when appropriate. Results were shown as mean ± S.E.

The studies performed on hPCLS were conducted on liver biopsies collected from two cirrhotic patients diagnosed with hepatitis C, and one patient with hepatocellular carcinoma, where the sample was obtained from the surrounding non-tumoral tissue. Figure 7 shows representative hematoxylin–eosin staining of the fibrotic and non-fibrotic tissue. Additionally, fibrosis quantification by Sirius Red demonstrated a significantly increased abundance of collagen fibers in the liver samples of patients with hepatitis C compared with the non-tumoral tissue of patients with hepatocellular carcinoma. The viability assessment of the hPCLS prepared from these samples demonstrated that both fibrotic and non-fibrotic samples maintained their viability for up to 72 h. Interestingly, fibrotic hPCLS had reduced ATP concentrations compared with non-fibrotic hPCLS, which is consistent with impaired metabolic activity in those samples. hPCLS from fibrotic livers also showed a significantly enhanced mRNA abundance of *COL1A1*, *αSMA*, and *MMP2*, reflecting more activated fibrogenic activity. This indicates that hPCLS maintain their molecular signature for at least 2 days.

## 3. Discussion

The present study aimed to assess the potential of PCLS as a platform for evaluating the efficacy of novel therapeutic interventions targeting liver fibrosis [7]. We first sought to investigate the effect of hyperoxic conditions on the viability, tissue integrity, and inflammatory response in PCLS from both control and fibrotic rats. While a lack of vascularization necessitates culturing PCLS under hyperoxic conditions to maintain tissue viability [8,9], there is no general agreement regarding the optimal O_2_ concentration that should be used. In previous studies, O_2_ values ranged between 20% and 95%, and these values also vary according to whether the tissue slices are from the lung, kidney, or liver. Focusing on liver slices, most studies were performed at concentrations ranging between 20% O_2_ and 80% O_2_ depending on the experimental design and objectives. This range is commonly selected to ensure the adequate oxygenation of the tissue while minimizing oxidative stress. For instance, 20% O_2_ has been used to approximate physiological conditions [13,14,15,16], whereas intermediate levels such as 40–60% have been applied to balance tissue viability and metabolic activity [13,15,17,18]. Higher concentrations, including 70–80% O_2_, are frequently employed to maintain viability in thicker slices or during prolonged incubations [19,20,21,22]. However, very little information is available regarding the effect of the different degrees of hyperoxia on tissue response. PCLS viability depends on a number of factors including the dimension of the PCLS (diameter and thickness), internal oxygenation, and media composition. These factors influence the ease of access of nutrients and oxygen to the inner cell layer, which in turn can influence the longevity of the PCLS cultured period. Our findings demonstrate that hyperoxia induces the expression of inflammatory mediators such as *Il6*, *Cox2*, and *Nos2.* Interestingly, the temporal regulation of *Il1b* has been described in various models of fibrosis, where IL1β signaling is most active during the initial immune response and diminishes as tissue remodeling advances [23,24]. This effect was observed in both the control and cirrhotic PCLS with the highest level of inflammatory response occurring at 80% O_2_. These results underscore the importance of carefully modulating O_2_ levels in ex vivo liver models to balance tissue viability and minimize inflammatory response. In this sense, the link between hyperoxia and oxidative stress is well established in the literature. Several studies have demonstrated that exposure to elevated oxygen levels leads to a reduction in intracellular GSH and an increase in oxidative stress markers [25,26]. Our current findings are consistent with previously reported mechanisms but require further validation in future studies with additional experiments.

In addition to examining the effects of hyperoxia, our study investigated the molecular signature of fibrotic PCLS and their response to an exogenous pro-inflammatory stimulus, such as LPS. Numerous studies have reported robust ECM remodeling in the livers of both humans and rodents with fibrosis [27,28,29,30]. This remodeling is accompanied by the upregulation of genes associated with fibrogenic and pro-inflammatory activity [31,32]. Our results demonstrate that fibrotic PCLS preserve their pathological gene expression profiles, characterized by elevated levels of *Col1α2*, *Col3α1*, and *Mmp2*, which are indicative of ongoing ECM remodeling. Moreover, the response of fibrotic PCLS to external stimuli mirrors that observed in fibrotic animal models [33]. Specifically, the exposure to LPS further amplified the pro-inflammatory response, as evidenced by the increased mRNA expression of *Nos2* and *Il1β*. In addition, the significant decrease in *Pparγ* expression following LPS exposure is consistent with previous studies showing that inflammatory stimuli can suppress *Pparγ* transcriptional activity, thereby promoting a pro-inflammatory and pro-fibrotic environment in liver tissue [34,35]. These findings suggest that fibrotic PCLS retain the molecular signature characteristic of the CCl_4_-induced fibrosis model, including the key features of inflammation and extracellular matrix remodeling. Future studies incorporating PCLS from alternative models of chronic liver disease, such as diet-induced or genetic models, will be essential to determine the extent to which disease-specific molecular and inflammatory signatures are preserved across different etiologies.

The time course analysis of mRNA expression in rPCLS demonstrated that fibrotic slices exhibited a sustained activation of fibrosis-related genes over a 48 h period. This prolonged activation highlights the stability of the fibrogenic phenotype in this ex vivo model, supporting its utility for investigating the progression of liver fibrosis and for preclinical testing of antifibrogenic therapies. Interestingly, and in contrast to earlier studies with rat liver slices [10,36], we did not observe the induction of the fibrogenic response in rPCLS from control rats after 48 h in culture. Notably, none of the profibrogenic genes, except *Mmp9*, showed significant activation over this period. This is likely related to the moderate O_2_ concentration at which our experiments were performed. To further validate the rPCLS model, we employed an adenoviral vector encoding a dominant negative form of *Pdgfrβ*. Previous studies by our laboratory and others have shown that the systemic administration of this kind of adenoviral construct in fibrotic rats results in the inhibition of hepatic stellate cell activation, reduced hepatic collagen deposition, increased mean arterial pressure, and reduced portal hypertension [7,37]. Based on these findings we assessed whether similar antifibrotic effects could be replicated in rPCLS. Indeed, infection with the adenoviral construct encoding the dominant-negative form of *Pdgfrβ* effectively reduced the expression of fibrosis-related genes in fibrotic rPCLS. In our experience with this experimental model, we have consistently observed that transcriptional changes in collagen-related genes occur rapidly and are detectable within 48 h. In contrast, the accumulation of collagen fibers at the tissue level typically requires a longer period to become histologically evident. This temporal dissociation has been documented in our laboratory during both the induction and resolution phases of fibrosis (unpublished data). These results reinforce the potential of PCLS as a robust platform for assessing the efficacy of novel therapeutic interventions targeting liver fibrosis.

Furthermore, our study extended to hPCLS derived from cirrhotic patients with hepatitis C and non-tumoral tissue from a patient with hepatocellular carcinoma. Our interest in assessing whether human fibrotic PCLS reproduce the molecular signature observed in fibrotic rPCLS resides in the already-described observation showing the differences in the fibrotic response between rPCLS and hPCLS [38], with underlying inter-species differences in the mechanisms of fibrosis. Although viability was maintained for the entire evaluation period in both types of hPCLS, significant differences were observed in the ATP concentrations between the two tissue types. Fibrotic hPCLS exhibited reduced ATP levels compared with non-fibrotic samples. This impaired metabolic activity in the fibrotic tissue is consistent with the impaired metabolic activity of the liver [39,40]. The fibrotic hPCLS also exhibited elevated mRNA levels of *COL1A1*, *αSMA,* and *MMP2*, reflecting an active fibrogenic state. Although an analysis of protein levels by Western blotting would have provided a more robust and consistent analysis of the mechanisms involved in the fibrogenic activity, this was not feasible due to the limited availability of human precision-cut liver slice (hPCLS) samples. In addition, the observed increase in COL1A1 and αSMA expression in non-fibrotic human PCLS may reflect species-specific differences and the sensitivity of human liver tissue to ex vivo culture conditions. Unlike rat PCLS, which are derived from young, healthy animals and tend to maintain a stable phenotype, human liver tissue may originate from donors with underlying metabolic or vascular conditions that predispose them to the low-grade activation of fibrogenic pathways, even in the absence of clinical fibrosis. This early activation may be further enhanced by the stress of ex vivo culture. These findings highlight the potential of this model for future therapeutic testing, based on its ability to preserve key fibrotic features ex vivo. hPCLS represents a promising platform for therapeutic testing, pending further validation with functional interventions, which are currently underway in our laboratory.

## 4. Materials and Methods

### 4.1. Induction of Liver Fibrosis in Rats

Studies were performed in 17 male adult Wistar rats (Charles-River, Saint Aubin les Elseuf, France). Rats with fibrosis (*n* = 12) and control rats (*n* = 5) were fed ad libitum with standard chow and water containing phenobarbital (0.3 g/L) as drinking fluid. The use of phenobarbital is not related to animal welfare, but rather to its pharmacological role as a hepatic enzyme inducer. Specifically, it enhances the activity of cytochrome P450 enzymes, particularly CYP2E1, which is responsible for the metabolic activation of CCl_4_ into its hepatotoxic intermediates. This co-administration is well established to potentiate the fibrogenic effects of CCl_4_ and ensure consistent fibrosis induction [41,42]. Fibrosis was induced by CCl_4_ inhalation for 17 weeks. Animals were exposed to a CCl_4_ vapor atmosphere twice a week, starting at 0.5 min per exposure. The duration of the exposure was increased by 1 min after every three sessions until it reached 5 min, which was used until the end of the investigation. Control rats were studied following a similar period of phenobarbital administration.

### 4.2. Rat Precision-Cut Liver Slices (rPCLS)

On the day of the experiment, fibrotic and control rats were sacrificed with an isofluorane overdose (Forane, Abbott Laboratories S.A., Madrid, Spain). A blood sample was obtained to measure standard liver function. The liver was excised and placed into ice-cold Storage Institut Georges Lopez-1 (IGL-1) Solution (IGL, Lissieu, France). Five mm diameter core biopsies were obtained and embedded in UltraPure low melting point agarose (3% (*w*/*v*), Invitrogen, Bleiswijk, The Netherlands) prewarmed at 37 °C. Once the agarose solidified, 250 µm thick precision-cut liver slices were prepared using the Leica VT1200 S vibratome (Leica Microsystems, Nussloch, Germany) filled with ice-cold Krebs–Henseleit buffer which consisted of 25 nM NaHCO_3_, 25 mM D-glucose, and 10 mM 4-(2-hydroxyethyl) piperazine-1-ethanesulfonic acid (HEPES) previously saturated with carbogen and adjusted to pH 7.4.

### 4.3. Incubation of the rPCLS

Slices were preincubated for 1 h in 12-well plates containing 1.3 mL of prewarmed and preoxygenated Williams Medium E (Gibco, Paisley, UK) supplemented with 50 µg/mL gentamycin (Gibco) and 25 mM D-glucose (Sigma-Aldrich, St. Louis, MO, USA) in a humidified atmosphere of 40% O_2_/5% CO_2_ or 80% O_2_/5% CO_2_ at 37 °C while gently shaken at 90 revolutions per minute. Freshly cut slices were included and considered as basal conditions. Slices were collected for further analysis after 24–72 h. In rPCLS treated with LPS (10 µg/mL), the exposure was applied during the first hour of culture and was limited to a single one-hour exposure. At least three slices were incubated for each condition. Total RNA was extracted as described above and gene expression assessed by RT-PCR.

### 4.4. Human Precision-Cut Liver Slices (hPCLS)

In brief, excess human liver was surgically obtained, and cylindrical cores were made using a 6 mm biopsy punch (Kai Medical, Seki City, Japan) and preserved in ice-cold IGL-1 preservation solution (Lissieu, France) until slicing. Krebs–Henseleit buffer was supplemented with 25 mM D-glucose (Merck, Darmstadt, Germany), 25 mM NaHCO3 (Merck), and 10 mM 4-(2-hydroxyethyl) piperazine-1-ethanesulfonic acid (Merck, Darmstadt, Germany), saturated with carbogen, and used as slicing solution. hPCLS were obtained using a Leica VT1200 S vibratome (Leica Microsystems, Nussloch, Germany). The size of the hPCLS was adjusted for the wet weight (4–5 mg) to a thickness of around 250 μm.

### 4.5. Incubation of the hPCLS

hPCLS were incubated as described previously [11]. William’s E medium with GlutaMAX (Gibco) 2.75 mg/mL D-glucose monohydrate (Merck, Darmstadt, Germany) and 50 µL/mL gentamicin (Gibco) was placed in 12-well plates (1.3 mL/well), preheated, and oxygenized in the incubator at 37 °C with a continuous 5% CO_2_–40% O_2_ supply for at least 30 min before plating the hPCLS. Freshly cut slices were included and considered as basal conditions. The hPCLS were preincubated individually for 1 h in the culture medium while gently shaken at 90 rev/min (basal time point). After preincubation, slices were changed to preheated and oxygenized fresh medium under different conditions. At the end of the experiment, triplicate slices for each condition of approximately 5 mg each were snap-frozen for measurements as previously described.

### 4.6. Assessment of Viability of rPCLS and hPCLS

After incubation, slices were collected individually in a 2 mL Eppendorf safe-lock tube with 1 mL sonification solution (2 mM EDTA Triplex in 70% ethanol at pH 10.9), snap-frozen, and stored at −80 °C until analysis. Slices were homogenized with 1 mL of sonication solution using a Miccra D-9 homogenizer (MICCRA GmbH, Heitersheim, Germany). PCLS viability was assessed by measuring the adenosine triphosphate (ATP) content in each slice using a bioluminescence assay kit class II (Roche Diagnostics, Mannheim, Germany) according to the manufacturer’s instructions. ATP content was normalized by measuring total protein concentrations in each slice using the Lowry method (DC Protein Assay, Bio-Rad, Veenendaal, The Netherlands).

### 4.7. Effect of Hyperoxia on mRNA Expression of Inflammatory Mediators in rPCLS

The study was performed in 250 μm thick rPCLS obtained from freshly harvested livers of control (*n* = 5) and cirrhotic Wistar rats (*n* = 9). First, rPCLS from the fibrotic rats included in the protocol were incubated in basal conditions and up to 48 h. under 5% CO_2_/40% O_2_ as previously described, and their viability was assessed using ATP measurement. Thereafter, the hepatic messenger expression of inflammatory genes was evaluated by real-time PCR.

### 4.8. Effect of a Dominant-Negative Soluble Platelet-Derived Growth Factor Receptor Beta (sPDGFRβ) on the Abrogation of PDGFRβ Signaling Pathway and the Messenger Expression of Profibrogenic Genes in rPCLS and hPCLS

The study was performed in 250 μm thick rPCLS obtained from freshly harvested livers of control (*n* = 5) and cirrhotic Wistar rats (*n* = 9) or from biopsies of surgically resected livers of cirrhotic (*n* = 2) and non-cirrhotic patients (*n* = 2). The rPCLS of fibrotic animals were randomly assigned to one of the following groups: A/rPCLS from 9 fibrotic rats were infected overnight with adenoviruses encoding for sPDGFRβ (Ad-sPDGFRβ) and B/rPCLS from 9 fibrotic rats were infected overnight with adenovirus encoding β-galactosidase (Ad-β-gal) as adenoviral control, at 1000 multiplicity of infection (m.o.i) in both cases. Following 48 h incubation (40% O_2_) tissue samples were collected to analyze tissue viability and hepatic expression of *Col1α2* and *Col3α1*.

### 4.9. Adenoviral Constructs

Two replication-defective recombinant adenoviral constructs, under the control of the cytomegalovirus promoter, expressing a dominant-negative soluble PDGFRβ (sPDGFRβ) encoding the extracellular domain of the PDGFRβ fused to the IgGFc domain of human immunoglobulin or β-gal were used [43,44]. All vectors were propagated in the HEK 293 cell line, and titers were determined by standard plaque assay [45].

### 4.10. Histological Examination

Liver sections (4 μm) were stained with H&E and Masson’s trichrome and digital images were acquired at 100× magnification with a microscope (Eclipse E600; Nikon, Tokyo, Japan) coupled to a digital camera (RT-Slider Spot; Diagnostic Instruments, Sterling Heights, MI, USA).

### 4.11. Hepatic Messenger Expression of Inflammatory, Cell Growth and Differentiation, and ECM Remodeling Genes in Fibrotic Rats

Total RNA was extracted from the middle liver lobe of control and fibrotic rats using a commercially available kit (RNAeasy; QIAGEN, Hilden, Germany). The RNA concentration was determined using spectrophotometric analysis (ND-100 spectrophotometer; Thermo Fisher Scientific, Waltham, MA, USA). One microgram of total RNA was reverse-transcribed using a cDNA synthesis kit (High-Capacity cDNA Reverse Transcription Kit; Applied Biosystems, Foster City, CA, USA). Specific primers and probes used for the different genes studied were designed to include intron spanning using the Universal ProbeLibrary Assay Design Center through ProbeFinder version 2.5 software (Roche Diagnostics, Indianapolis, IN, USA; https://primers.neoformit.com (accessed on 5 June 2022)). A panel of selected genes dealing with inflammation, cell growth and differentiation, and extracellular matrix remodeling activity was analyzed. The inflammation panel included the following: interleukin 1 beta (*IL1β*) (probe#76: left 5′-CAGGAAGGCAGTGTCACTCA-3′ and right 5′-TCCCACGAGTCACAGAGGA-3′), interleukin 6 (*IL6*) (probe#20: left 5′-CCCTTCAGGAACAGCTATGAA-3′ and right 5′-ACAACATCAGTCCCAAGAAGG-3′), tumor necrosis factor alpha (*TNFα*) (probe#68: left 5′-CGTAGCCCACGTCGTAGC-3′ and right 5′-GGTTGTCTTTGAGATCCATGC-3′), inducible Nitric Oxide Synthase (*Nos2*) (probe#95: left 5′-AAAATGGTTTCCCCCAGTTC-3′ and right 5′-CAGCTTGTCCAGGGATTCTG-3′), and cyclooxygenase 2 (*Cox2*) (probe#125: left 5′-GATGCTATCTTTGGGGAGACC-3′ and right 5′-CCATAAGGCCTTTCAAGGAGA-3′). The extracellular matrix remodeling panel included the following: platelet-derived growth factor receptor β (*Pdgfrβ*) (probe#69; left 5′-GCGGAAGCGCATCTATATCT-3′, right 5′-GCGGAAGCGCATCTATATCT-3′), transforming growth factor β receptor 1 (*Tgfβr1*) (probe#53; left 5′-AAGGCCAAATATTCCCAACA-3′, right 5′-ATTTTGGCCATCACTCTCAAG-3′), Collagen Iα2 (*Col1α2*) (probe#95; left 5′-AGACCTGGCGAGAGAGGAGT-3′, right 5′-ATCCAGACCGTTGTGTCCTC-3′), Collagen IIIα1 (*Col3α1*) (probe#49; left 5′-TCCCCTGGAATCTGTGAATC-3′, right 5′-TGAGTCGAATTGGGGAGAAT-3′), α-smooth muscle actin (*αSMA*) (probe#78; left 5′-CATCAGGAACCTCGAGAAGC-3′, right 5′-AGCCATTGTCACACACCAGA-3′), tissue inhibitor of matrix metalloproteinases type 1 (*Timp1*) (probe#95; left 5′-CATGGAGAGCCTCTGTGGAT-3′, right 5′-TGTGCAAATTTCCGTTCCTT-3′), tissue inhibitor of matrix metalloproteinases type 2 (*Timp2*) (probe#73; left 5′-GACAAGGACATCGAATTTATCTACAC-3′, right 5′-CCATCTCCTTCCGCCTTC-3′), matrix metalloproteinases type 2 (*Mmp2)* (probe#60; left 5′-CTCCACTACGCTTTTCTCGAAT-3′, right 5′-TGGGTATCCATCTCCATGCT-3′), and matrix metalloproteinases type 9 (*Mmp9)* (probe#53; left 5′-CCTGAAAACCTCCAACCTCA-3′, right: 5′-GAGTGTAACCATAGCGGTACAGG-3′). Hypoxanthine-guanine phosphoribosyltransferase (*Hprt*) (probe#95: left 5′-GACCGGTTCTGTCATGTCG-3′, right 5′-ACCTGGTTCATCATCACTAATCAC-3′ was used as the reference gene. Primers were designed according to rat sequences (GenBank codes NM_031512.2, NM_012589.1, NM_012675.3, NM_012611.3, NM_017232.3, NM_031525.1, NM_012775.2, NM_053356.1, NM_032085.1, NM_031004.2, NM_053819.1, NM_021989.2, NM_031054.2, NM_031055.1, and NM_012583.2). A real-time quantitative polymerase chain reaction was analyzed in duplicate and performed with the LightCycler 480 (Roche Diagnostics). A 10 µL total volume reaction of diluted 1:8 cDNA, 200 nM primer dilution, 100 nM prevalidated 9-mer probe (Universal ProbeLibrary, Roche), and FastStart TaqMan Probe Master (Roche Diagnostics) were used in each polymerase chain reaction. A fluorescence signal was captured during each of the 45 cycles (denaturizing for 10 s at 95 °C, annealing for 20 s at 60 °C, and extension for 1 s at 72 °C). Water was used as a negative control. Relative quantification was calculated using the comparative threshold cycle (CT), which is inversely related to the abundance of mRNA transcripts in the initial sample. The mean CT of duplicate measurements was used to calculate ΔCT as the difference in CT for target and reference. The relative quantity of product was expressed as the fold induction of the target gene compared with the reference gene according to the formula 2^−ΔΔCT^, where ΔΔCT represents ΔCT values normalized with the mean ΔCT of control samples.

### 4.12. Other Measurements

Standard serum biochemistry parameters of liver function were measured in the BS-200E Chemistry Analyzer (Mindray Medical International Ltd., Shenzhen, China).

### 4.13. Statistical Analysis

Data were analyzed using GraphPad Prism 6 (GraphPad Software Inc., San Diego, CA, USA). One-way ANOVA and Tukey’s multiple comparison test or Kruskal–Wallis’s and Dunn’s multiple comparison tests were used when appropriate, and unpaired Student’s *t*-test or Mann–Whitney’s test were used when appropriate. Results were shown as mean ± S.E. and considered significant when *p* < 0.05.

## 5. Conclusions

Our study demonstrates that PCLS both from rat and human origins are valuable ex vivo models for investigating liver fibrosis. The ability to maintain tissue viability and pathological gene expression profiles under moderate hyperoxic conditions along with the responsiveness to inflammatory stimuli and therapeutic intervention underscore the utility of PCLS in translational liver research.

## Figures and Tables

**Table 1:** Serum markers of liver function in control (*n* = 5) and fibrotic (*n* = 12) rPCLS donor animals included in the different protocols.

	Control	Fibrosis
AST (U/L)	123.2 ± 15.50	561.20 ± 96.14 **
ALT (U/L)	45.12 ± 3.78	264.10 ± 77.53 ***
GGT (U/LL)	0 ± 0	6.85 ± 2.10 ***
LDH (U/L)	618.1 ± 61.67	1286 ± 913.50 *
T-Bilirubin (mg/dL)	0.12 ± 0.02	1.27 ± 0.32 *
Glucose (mg/dL)	190.0 ± 16.47	125.4 ± 6.76 ***
Total proteins (g/L)	69.24 ± 2.39	57.43 ± 1.88 **
Albumin (g/L)	34.98 ± 2.34	28.72 ± 1.36 *
Triglycerides (mg/dL)	134.4 ± 32.42	59.56 ± 5.80 **
Total cholesterol (mg/dL)	78.73 ± 14.49	89.22 ± 4.51
Serum Na^+^ (mEq/L)	141.1 ± 0.48	139.4 ± 0.73
Serum K^+^ (mEq/L)	4.24 ± 0.22	4.47 ± 0.08

*** *p* < 0.001, ** *p* < 0.01, * *p* < 0.05 vs. Control. Unpaired *t*-test or Mann–Whitney when appropriate.

**Table 2:** Viability and hepatic mRNA expression of genes related to extracellular matrix remodeling and inflammation in PCLS of control rats (*n* = 4) and cirrhotic rats (*n* = 4) after 1 h incubation.

	Control	Cirrhosis	*p* Value
Tissular viability			
ATP (pmol/μg prot)	11.8 ± 1.4	8.3 ± 0.5	<0.05
Extracellular Matrix Remodeling			
*Col1α2*	1 ± 0.2	12.01 ± 3.6	<0.05
*Col3α1*	1 ± 0.1	7.4 ± 2.2	<0.01
*αSMA*	1 ± 0.2	3.8 ± 0.7	<0.05
*Timp1*	1 ± 0.1	4.5 ± 1.0	<0.05
*Timp2*	1 ± 0.2	2.6 ± 0.4	<0.05
*Mmp2*	1 ± 0.1	9.9 ± 2.7	<0.05
*Mmp9*	1 ± 0.3	1.2 ± 0.2	NS
Inflammation and Growth factors			
*Pdgfrβ*	1 ± 0.2	4.1 ± 1.2	<0.01
*Tgfβr1*	1 ± 0.2	0.9 ± 0.1	NS
*Nos2*	1 ± 0.7	8.1 ± 4.3	<0.05
*Cox2*	1 ± 0.3	6.9 ± 2.5	<0.05
*Il1β*	1 ± 0.3	1.9 ± 0.4	NS
*Tnfα*	1 ± 0.1	0.6 ± 0.2	NS
*Il6*	0.9 ± 0.3	13.8 ± 4.7	<0.05

*Col1α2*, Collagen Type I Alpha 2 Chain; *Col3α1*, Collagen Type III Alpha 1 Chain; *αSMA*, Alpha Smooth Muscle Actin; *Timp1*, tissue inhibitor of metalloproteinases 1; *Timp2*, tissue inhibitor of metalloproteinases 2; *Mmp2*, metalloproteinase 2; *Mmp9*, metalloproteinase 9; *Pdgfrβ*, platelet-derived growth factor receptor beta; *Tgfβr1*, transforming growth factor receptor beta; *Nos2*, nitric oxide synthase 2; *Cox2*, cyclooxygenase 2; *Il1β*, interleukin 1 beta; *Tnfα*, tumor necrosis factor alpha; *Il6*, interleukin 6. Unpaired Student’s *t*-test or Mann–Whitney’s test were used when appropriate. NS, non-significant. Results are given as means ± S.E.

## Data Availability

The data presented in this study are available within the article.

## Abbreviations

The following abbreviations are used in this manuscript:

ATP	Adenosine triphosphate
Ad-sPDGFRβ	Adenoviruses encoding for sPDGFRβ
*αSMA*	α-smooth muscle actin
CCl_4_	Carbon tetrachloride
*Col1α2*	Collagen Iα2
*Col3α1*	Collagen IIIα1
*Cox2*	Cyclooxygenase 2
*sPDGFRβ*	Dominant-negative soluble platelet-derived growth factor receptor beta
ECM	Extracellular matrix
HSC	Hepatic stellate cells
HEPES	4-(2-hydroxyethyl) Piperazine-1-ethanesulfonic acid
*Hprt*	Hypoxanthine-guanine phosphoribosyltransferase
*Nos2*	Inducible Nitric Oxide Synthase
IGL-1	Institut Georges Lopez-1
*IL1β*	Interleukin 1 beta
*IL6*	Interleukin 6
LPS	Lipopolysaccharide
*Mmp2*	Matrix metalloproteinases type 2
*Mmp9*	Matrix metalloproteinases type 9
MASLD	Metabolic dysfunction-associated steatotic liver disease
*PDGF*	Platelet-derived growth factor
*Pdgfrβ*	Platelet-derived growth factor receptor β
PCLS	Precision-cut liver slices
ROS	Reactive oxygen species
*Timp1*	Tissue inhibitor of matrix metalloproteinases type 1
*Timp2*	Tissue inhibitor of matrix metalloproteinases type 2
*TGFβ*	Transforming growth factor-β
*Tgfβr1*	Transforming growth factor β receptor 1
*TNFα*	Tumor necrosis factor alpha
